# Association of kinesin family member 2A with increased disease risk, deteriorative clinical characteristics, and shorter survival profiles in acute myeloid leukemia

**DOI:** 10.1590/1414-431X20209173

**Published:** 2020-12-18

**Authors:** Tianling Ding, Jialing Li, Jianhong Sun, Xiaoman Fan, Chunli Shi, Dong Zhou, Ruoyu Deng

**Affiliations:** 1 Department of Hematology, Huashan Hospital, Fudan University, Shanghai, China; 2 Department of Hematology, Huashan Hospital North, Fudan University, Shanghai, China; 3 Shanghai Qeejen Bio-tech Institution, Shanghai, China

**Keywords:** Acute myeloid leukemia, Cell apoptosis, Clinical characteristics, Kinesin family member 2A, Survival

## Abstract

This study aimed to explore the correlation of kinesin family member 2A (KIF2A) expression with disease risk, clinical characteristics, and prognosis of acute myeloid leukemia (AML), and investigate the effect of KIF2A knockdown on AML cell activities *in vitro*. Bone marrow samples were collected from 176 AML patients and 40 healthy donors, and KIF2A expression was measured by real-time quantitative polymerase chain reaction. Treatment response, event-free survival (EFS), and overall survival (OS) were assessed in AML patients. *In vitro*, KIF2A expression in AML cell lines and CD34^+^ cells (from healthy donors) was measured, and the effect of KIF2A knockdown on AML cell proliferation and apoptosis in HL-60 and KG-1 cells was detected. KIF2A expression was greater in AML patients compared to healthy donors, and receiver operating characteristic curve indicated that KIF2A expression predicted increased AML risk (area under curve: 0.793 (95%CI: 0.724-0.826)). In AML patients, KIF2A expression positively correlated with white blood cells, monosomal karyotype, and high risk stratification. Furthermore, no correlation of KIF2A expression with complete remission or hematopoietic stem cell transplantation was found. Kaplan-Meier curves showed that KIF2A expression was negatively correlated with EFS and OS. *In vitro* experiments showed that KIF2A was overexpressed in AML cell lines (KG-1, HL-60, ME-1, and HT-93) compared to CD34^+^ cells, moreover, cell proliferation was reduced but apoptosis was increased by KIF2A knockdown in HL-60 and KG-1 cells. In conclusion, KIF2A showed potential to be a biomarker and treatment target in AML.

## Introduction

Acute myeloid leukemia (AML), one of the most malignant hematopoietic system diseases, is characterized by abnormal proliferation, poor cell differentiation, and infiltration of bone marrow, peripheral blood, or other tissues (1,2). With the advances in treatment for AML, the response rates after initial chemotherapy increased in adult AML patients, while 20-70% of patients relapse after achieving complete remission (CR), resulting in a poor prognosis of AML patients with a low five-year survival (less than 30%) (1,3). Emerging evidence has suggested that biomarkers related to disease progression and prognosis would contribute to individualize therapy and improve prevention of aggressive disease or treatment outcomes in AML patients.

Kinesin superfamily proteins (KIFs) are microtubule-dependent molecular motor proteins presenting adenosine triphosphatase activity (4). Kinesin family member 2A (KIF2A), a member of kinesin-13 family, is essential in assembling normal bipolar spindles and mitosis progression (5,6). A few studies have shown that KIF2A is overexpressed in tumor tissues and correlates with clinicopathological features in patients with some cancers (such as diffuse large B cell lymphoma (DLBCL), breast cancer, and lung squamous cell carcinoma) (7–9). Furthermore, some *in vitro* experiments have shown that KIF2A functions as a tumor oncogene in several cancer cell lines (such as human malignant glioma, lung adenocarcinoma, and squamous cell carcinoma of the oral tongue) (4,10,11). Based on these implications about the tumor-promotive effect of KIF2A in various cancers, we speculated that KIF2A might also play a crucial role in disease progression and underlying mechanisms of AML, whereas relevant evidence is rarely reported. Hence, this study aimed to explore the correlation of KIF2A expression with AML risk and to investigate the correlation of its expression with clinical characteristics, event-free survival (EFS), as well as overall survival (OS) of AML patients. Moreover, the effect of KIF2A knockdown on cell activities *in vitro* was explored.

## Material and Methods

### Subjects

From January 2015 to March 2019, 176 *de novo* AML patients treated at Huashan Hospital were consecutively recruited. The eligibility for inclusion consisted of: i) confirmed diagnosis of primary AML in accordance with the World Health Organization (WHO) Classification of Tumors of Hematopoietic and Lymphoid Tissues (2008); ii) age ≥18 years; and iii) no systematic treatment for AML before enrollment. The exclusion criteria included: i) secondary AML; ii) acute promyelocytic leukemia; iii) accompanied with other malignancies; iii) history of radiotherapy, chemotherapy, or allogeneic stem cell transplantation; iv) infected with human immunodeficiency virus; and v) pregnant or lactating women. During the same period, 40 healthy bone marrow (BM) donors were enrolled as healthy controls when undergoing donation of BM at our hospital.

### Ethics statement

The Ethics Committee of Huashan Hospital approved this study before its initiation. The study was carried out in accordance with the principles expressed in the Declaration of Helsinki. All subjects provided written informed consent before recruitment.

### Clinical data collection

Clinical data of AML patients were collected on enrollment, which consisted of demographic characteristics (age and gender), clinical features (French-American-British (FAB) classification, cytogenetics, molecular genetics, and risk stratification), and laboratory test (white blood cells (WBC)). The FAB classification was assessed in accordance with criteria developed by the FAB Cooperative Group (12), and risk stratification was evaluated according to National Comprehensive Cancer Network (NCCN) Clinical Practice Guidelines in Oncology of AML (Version 2.2013).

### Sample collection and detection

BM samples of AML patients were extracted before initiation of therapy, and the BM samples of healthy controls were collected on BM donation. After collection, BM mononuclear cells (BMMCs) were isolated from BM samples, and CD34^+^ cells were further isolated from BMMCs of healthy controls. The relative expression of KIF2A mRNA was determined using real-time quantitative polymerase chain reaction (RT-qPCR).

### Treatment and follow-up

Conventional induction therapy was performed on the basis of clinical status of patients after the diagnostic work-up had been completed, which was administered according to the AML guidelines. Following conventional induction therapy with 3 days of anthracycline and 7 days of cytarabine (“3+7”) or therapies of comparable intensity, response assessment was commonly carried out between days 21 and 28 after initiation of therapy. CR was defined as follows (all criteria need to be fulfilled): bone marrow blasts <5%, absence of blasts with Auer rods, absence of extramedullary disease, absolute neutrophil count >1.0×10^9^/L (1,000/μL), platelet count >100×10^9^/L (100,000/μL), and independence of red cell transfusions (13). After induction therapy, the following therapy was based on the remission status and risk classification, including intensive conventional chemotherapy, prolonged maintenance treatment, and high-dose therapy followed by autologous or allogeneic hematopoietic stem cell transplantation (HSCT). In addition, surveillance and follow-up were performed every 3 to 6 months or as clinically indicated. Patients in this study were followed-up until March 31, 2019, with a median follow-up duration of 17.0 months and a total follow-up duration ranging from 1.0 to 47.0 months. OS was calculated from the date of entry into the study to the date of death from any cause, and patients not known to have died at last follow-up were censored on the date they were last known to be alive. EFS was calculated from the date of entry into the study to the date of induction treatment failure, or relapse from CR, or death from any cause, and patients not known to have any of these events were censored on the date they were last examined.

### Cell culture

Human AML cell lines KG-1, HL-60, ME-1, and HT-93 were purchased from Leibniz Institute DSMZ-German Collection of Microorganisms and Cell Cultures (Germany) and cultured in 90% Roswell Park Memorial Institute (RPMI) 1640 medium (Gibco, USA) with 10% fetal bovine serum (Gibco). All cells were contained in a humidified atmosphere of 95% air and 5% CO_2_ at 37°C.

### Detection of KIF2A in AML cell lines

To detect the expressions of KIF2A mRNA and protein in KG-1, HL-60, ME-1, and HT-93 cell lines, RT-qPCR and western blot were performed, with the healthy donors' BMMCs used as control.

### Transfection and detection

The knockdown KIF2A plasmid and the knockdown control plasmid were constructed with the use of pRNAT-U6.1/Neo vector (Guangzhou RiboBio Co., Ltd., China). The constructed plasmids were transfected into HL-60 and KG-1 cells using HilyMax (Dojindo, Japan), and the shRNA sequences used for KIF2A inhibition were: forward, 5′-CACCGCTGAAGAAGCCAAACTATCGAAATAGTTTGGCTTCTTCAGC-3′; reverse, 5′-AAAAGCTGAAGAAGCCAAACTATTTCGATAGTTTGGCTTCTTCAGC-3′.

Notably, we used three shRNAs to knockdown KIF2A, and we chose the one that had the best efficiency for KIF2A knockdown for the following assays. The cells transfected with knockdown KIF2A plasmid were defined as KIF2A-KD group, and the cells transfected with knockdown control plasmid were defined as negative control (NC)-KD group, accordingly. At 24 h after transfection, the mRNA and protein expressions of KIF2A in cells were detected by RT-qPCR and western blot. Cell proliferation and cell apoptosis rate were detected by Cell Counting kit (CCK-8) and Annexin V (AV)/Propidium Iodide (PI) assay. The CCK-8 assay was performed at 0, 24, 48, and 72 h after transfection, and the AV/PI assay was carried out 48 h after transfection.

### RT-qPCR

For RT-PCR assay, PureZOL RNA isolation reagent (BioRad, USA) was used to extract the total RNA, and PrimeScript™ RT Master Mix (Takara, Japan) was used to conduct the cDNA transcription. Subsequently, qPCR was performed by TB Green™ Fast qPCR mix (Takara, Japan), and the final results were calculated by the 2^−△△CT^ formula. GAPDH was used as the internal reference. Primers used were as follows: KIF2A, forward primer: 5′ GCCGAATACATCAAGCAATGGT 3′, reverse primer: 5′ TGCTGGAGGTGGAGGTGTT 3′; GAPDH, forward primer: 5′ GAGTCCACTGGCGTCTTCAC 3′, reverse primer: 5′ ATCTTGAGGCTGTTGTCATACTTCT 3′.

### Western blot

For western blot assay, total protein was extracted using RIPA buffer (Sigma, USA), and the protein concentration was determined by the Bicinchoninic Acid Kit for Protein Determination (Sigma, USA). For protein electrophoresis, NuPAGE™ 4-12% Bis-Tris protein gels (Invitrogen, USA) were used, and then proteins were transferred to PVDF membrane (Millipore, Germany), which was further incubated with primary antibody [Rabbit polyclonal to KIF2A (1:2000, Abcam, UK) and Rabbit-anti-GAPDH antibody (1:10000, Abcam)], and secondary antibody [Goat Anti-Rabbit IgG H&L (HRP) (1:5000, Abcam)], in turn. Furthermore, the bands were visualized by Pierce™ECL Plus Western Blotting substrate (Thermo, USA) and X-ray film (Kodak, USA).

### CCK-8 assay

Culture medium was discarded and cells were washed. Then, Cell Counting kit-8 (Sangon, China) mixed with fresh culture medium was added to the cells. Subsequently, cells were incubated for 2 h under 95% air plus 5% CO_2_ at 37°C. Absorbance was read by a microplate reader (Biotek, USA), which represented cell proliferation.

### AV/PI assay

At 48 h post-transfection, cells were prepared as suspension. Annexin V-FITC Apoptosis Detection kit (Sigma, USA) was used to perform AV/PI assay according to the instructions of the manufacturer, and finally, the apoptosis rate was assessed.

### Statistical analysis

Statistical analyses were carried out using SPSS 21.0 statistical software (IBM, USA) and GraphPad Prism 7.02 statistical software (GraphPad Software Inc., USA). Continuous variables are reported as means±SD or median and inter-quartile range (IQR); categorized variables are reported as count (percentage). Differences were compared with the independent samples *t*-test, chi-squared test, Wilcoxon rank-sum test, or one-way analysis of variance (ANOVA) followed by Dunnett *t*-test, as appropriate. Receiver operating characteristic (ROC) curve analysis and the derived area under curve (AUC) were used to assess the feasibility of variable to distinguish different subjects. Kaplan-Meier (K-M) curves were plotted to display the EFS and OS of subgroups, and the differences of EFS and OS between subgroups were determined by the log-rank test. Analyses of factors affecting EFS and OS were assessed by univariate and multivariate Cox proportional hazard regression model analyses. All the experiments were carried out in triplicate. P values <0.05 were considered statistically significant.

## Results

### Baseline characteristics

One hundred and seventy-six (90 males and 86 females) AML patients were enrolled in our study with a mean age of 44.4±12.2 years and median age of 44.0 (35.0-54.0) years. Further details are shown in Table 1.

**Table 1:** Characteristics of acute myeloid leukemia (AML) patients.

Items	AML patients (N=176)
Age (years)	
Mean±SD	44.4±12.2
Median (IQR)	44.0 (35.0-54.0)
Gender (male/female), n	90/86
WBC (*10^9^/L)	
Mean±SD	21.1±16.1
Median (IQR)	19.0 (7.3-30.7)
≤10*10^9^/L, n (%)	57 (32.4)
>10*10^9^/L, n (%)	119 (67.6)
FAB classification, n (%)	
M1	1 (0.6)
M2	63 (35.8)
M4	44 (25.0)
M5	60 (34.1)
M6	8 (4.5)
Cytogenetics, n (%)	
NK	79 (44.9)
inv(16) or t(16;16)	19 (10.8)
CK	14 (7.9)
t(8;21)	13 (7.4)
+8	11 (6.2)
-7 or 7q-	7 (4.0)
t(9;11)	5 (2.8)
-5 or 5q-	4 (2.3)
t(9;22)	3 (1.7)
11q23	2 (1.1)
inv(3) or t(3;3)	1 (0.6)
t(6;9)	1 (0.6)
Others (not included in low or high risk)	17 (9.7)
Monosomal karyotype, n (%)	17 (9.7)
FLT3-ITD mutation, n (%)	39 (22.2)
Isolated biallelic CEBPA mutation, n (%)	19 (10.8)
NPM1 mutation, n (%)	64 (36.4)
Risk stratification, n (%)	
Low	55 (31.2)
Intermediate	63 (35.8)
High	58 (33.0)

IQR: interquartile range; WBC: white blood cells; FAB classification: French-American-Britain classification; NK: normal karyotype; CK: complex karyotype; FLT3-ITD: internal tandem duplications in the FMS-like tyrosine kinase 3; CEBPA: CCAAT/enhancer-binding protein α; NPM1: nucleophosmin 1.

### KIF2A expression in AML patients

**Figure 1:**
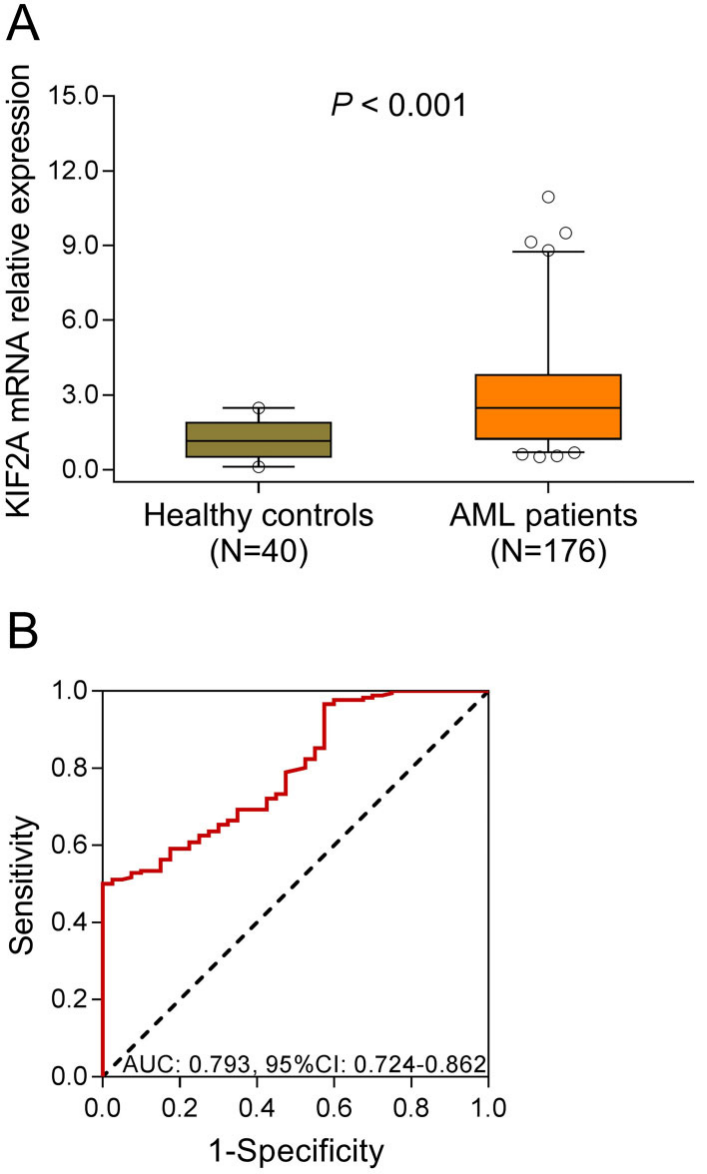
A, KIF2A mRNA expression in acute myeloid leukemia (AML) patients and healthy controls. **B**, ROC curve of KIF2A distinguishing AML patients from healthy controls. Data are reported as median and inter-quartile range. Comparison was determined by Wilcoxon rank-sum test. P<0.05 was considered significant. KIF2A: kinesin family member 2A; ROC curve: receiver operating characteristic curve; AUC: area under the curve; CI: confidence interval.

KIF2A expression was elevated in AML patients [2.480 (1.232-3.809)] compared to healthy controls [1.162 (0.515-1.878)] (P<0.001) (Figure 1A), and ROC curve showed that KIF2A expression was able to distinguish AML patients from healthy controls with AUC of 0.793 (95%CI: 0.724-0.826) (Figure 1B). Moreover, according to the analyses published on GEO database (https://www.ncbi.nlm.nih.gov/geoprofiles), we found two datasets that exhibited KIF2A expressions in AML patients and normal controls (Figure S1): in detail, one dataset showed that KIF2A expression was increased in the bone marrow samples of pediatric AML patients compared to that of normal controls (Figure S1A), and the other one showed that KIF2A expression was similar between the bone samples of AML patients and those of normal controls (Figure S1B). These data corroborated our primary observation of higher KIF2A expression in AML patients.

### Correlation of KIF2A expression with clinical characteristics in AML patients

KIF2A high expression was correlated with increased WBC level (P=0.036), monosomal karyotype (P=0.022), and high risk stratification in AML patients (P=0.001) (Table 2). Furthermore, KIF2A high expression was numerically associated with FAB classification M2, but without statistical significance (P=0.084). However, no correlation of KIF2A expression with other clinical characteristics including age (P=0.544), gender (P=0.132), FAB classification M1 (P=1.000), FAB classification M4 (P=0.486), FAB classification M5 (P=0.340), FAB classification M6 (P=1.000), cytogenetics (P=0.880), FLT3-ITD mutation (P=0.204), isolated biallelic CEBPA mutation (P=0.808), and NPM1 mutation (P=0.210) was found in AML patients.

**Table 2:** Correlation of KIF2A mRNA relative expression with clinical characteristics.

Items	KIF2A mRNA relative expression		P value
	High	Low	
Age, n (%)			0.544
≤45 years	47 (48.0)	51 (52.0)	
>45 years	41 (52.6)	37 (47.4)	
Gender, n (%)			0.132
Male	50 (55.6)	40 (44.4)	
Female	38 (44.2)	48 (55.8)	
WBC, n (%)			0.036
≤10*10^9^/L	22 (38.6)	35 (61.4)	
>10*10^9^/L	66 (55.5)	53 (44.5)	
FAB classification, n (%)			
M1	1 (100.0)	0 (0.0)	1.000
M2	26 (41.3)	37 (58.7)	0.084
M4	24 (54.5)	20 (45.5)	0.486
M5	33 (55.0)	27 (45.0)	0.340
M6	4 (50.0)	4 (50.0)	1.000
Cytogenetics, n (%)			0.880
NK	39 (49.4)	40 (50.6)	
Others*	49 (50.5)	48 (49.5)	
Monosomal karyotype, n (%)			0.022
No	75 (47.2)	84 (52.8)	
Yes	13 (76.5)	4 (23.5)	
FLT3-ITD mutation, n (%)			0.204
No	65 (47.4)	72 (52.6)	
Yes	23 (59.0)	16 (41.0)	
Isolated biallelic CEBPA mutation, n (%)			0.808
No	78 (49.7)	79 (50.3)	
Yes	10 (52.6)	9 (47.4)	
NPM1 mutation, n (%)			0.210
No	60 (53.6)	52 (46.4)	
Yes	28 (43.8)	36 (56.3)	
Risk stratification, n (%)			0.001
Low	16 (29.1)	39 (70.9)	
Intermediate	36 (57.1)	27 (42.9)	
High	36 (62.1)	22 (37.9)	

Comparison was determined by chi-squared test, Fisher's exact test, or Wilcoxon rank sum test. High or low relative expression of KIF2A mRNA was classified by median value. KIF2A: kinesin family member 2A; WBC: white blood cells; FAB classification: French-American-Britain classification systems; NK: normal karyotype; FLT3-ITD: internal tandem duplications in the FMS-like tyrosine kinase 3; CEBPA: CCAAT/enhancer-binding protein α; NPM1: nucleophosmin 1; CK: complex karyotype. *included inv(16) or t(16;16), CK, t(8;21), +8, -7 or 7q-, t(9;11), -5 or 5q-, t(9;22), 11q23, inv(3) or t(3;3), t(6;9), and those that were not included in low or high risk.

### Correlation of KIF2A expression with CR and HSCT in AML patients

**Figure 2:**
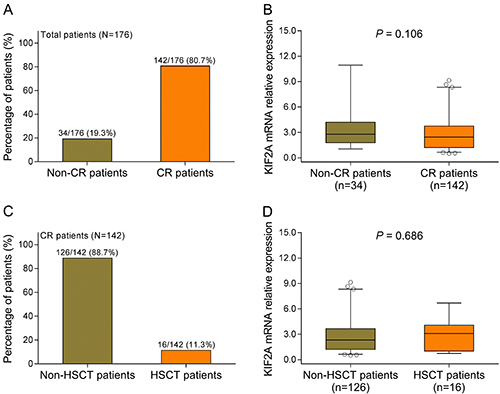
A, Percentages of patients that achieved complete remission (CR) and patients that did not achieve CR. **B**, KIF2A mRNA expression in CR patients and non-CR patients. **C**, Percentages of patients that received, or not, hematopoietic stem cell transplantation (HSCT). **D**, KIF2A mRNA expression in HSCT patients and non-HSCT patients. Data are reported as median and inter-quartile range. P<0.05 was considered significant (chi-squared test). KIF2A: kinesin family member 2A; mRNA: messenger RNA.

A total of 142 (80.7%) patients achieved CR while 34 (19.3%) did not achieve CR, and there was a trend of decreased KIF2A expression in CR patients compared to non-CR patients but without statistical significance (P=0.106) (Figure 2A and B). Among the CR patients, 16 (11.3%) and 126 (88.7%), respectively, received HSCT and did not receive HSCT, meanwhile, no correlation of KIF2A expression with HSCT was found (P=0.686) (Figure 2C and D).

### Correlation of KIF2A expression with survival profiles in AML patients

**Figure 3:**
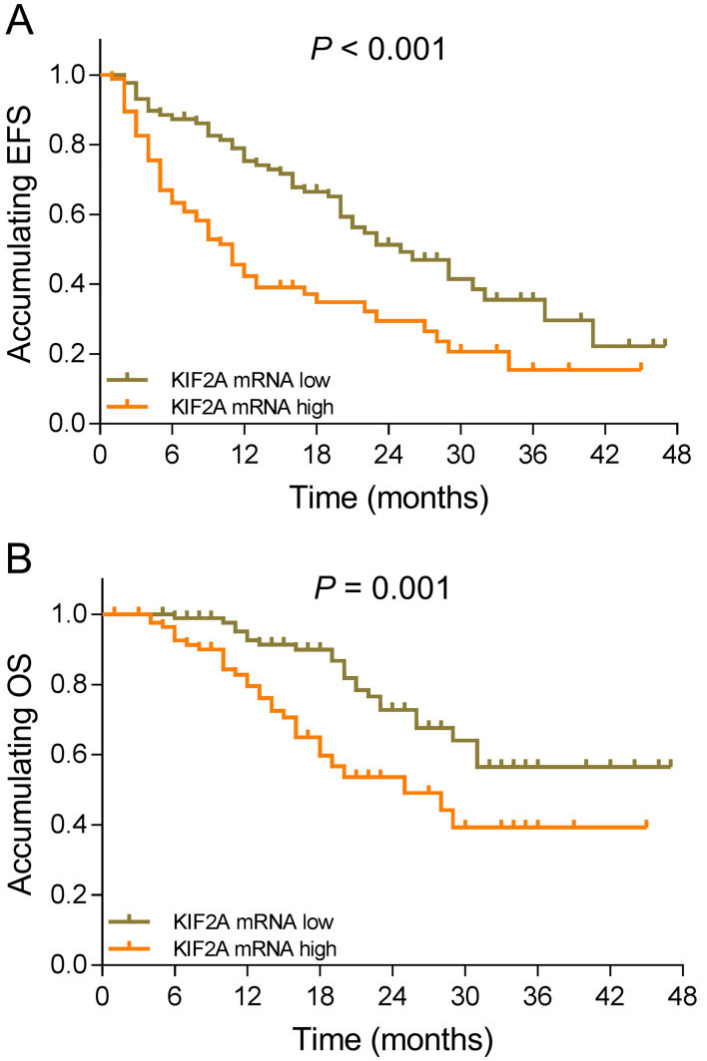
A, Event-free survival (EFS) in KIF2A high expression patients and KIF2A low expression patients. **B**, Overall survival (OS) in KIF2A high expression patients and KIF2A low expression patients. OS is reported by Kaplan-Meier curves. P<0.05 was considered significant (log-rank test). KIF2A: kinesin family member 2A.

KIF2A high expression was associated with shorter EFS (P<0.001) (Figure 3A) and decreased OS in AML patients (P=0.001) (Figure 3B). Additionally, all patients were categorized as subgroups according to risk stratification and correlation of EFS as well as OS when risk stratification was performed. As shown in Figure S2A, increased risk stratification was associated with reduced EFS (P<0.001), and KIF2A expression was negatively correlated with EFS in intermediate risk patients (P=0.049) (Figure S2C), while no correlation of KIF2A expression with EFS was found in low risk patients (P=0.673) (Figure S2B) or high risk patients (P=0.203) (Figure S2D). Furthermore, higher risk stratification was associated with worse OS (P<0.001) (Figure S2E), and KIF2A expression was negatively correlated with OS in high risk patients (P=0.091) (Figure S2H), but without statistical significance, and no correlation of KIF2A expression with OS was observed in low risk patients (P=0.562) (Figure S2F) or intermediate risk patients (P=0.403) (Figure S2G).

### Analysis of factors affecting survival profiles in AML patients

In addition, univariate Cox proportional hazards regression model analysis showed that KIF2A high expression (P<0.001), WBC >10×10^9^/L (P=0.002), monosomal karyotype (P<0.001), FLT3-ITD mutation (P=0.008), and high risk stratification were associated with shorter EFS; multivariate Cox proportional hazards regression model analysis displayed that WBC >10×10^9^/L (P=0.000) and high risk stratification were independent predictive factors for shorter EFS in AML patients (Table 3). Univariate Cox proportional hazards regression model analysis indicated that KIF2A high expression (P=0.002), WBC >10×10^9^/L (P=0.044), monosomal karyotype (P<0.001), NPM1 mutation (P=0.046), and high risk stratification were associated with worse OS and multivariate Cox proportional hazards regression model analysis showed that WBC >10×10^9^/L (P=0.002) and high risk stratification were independent factors predicting worse OS in AML patients (Table 4).

**Table 3:** Factors affecting event-free survival by Cox proportional hazards regression model analyses.

Parameters	Univariate Cox regression				Multivariate Cox regression			
	P value	HR	95%CI		P value	HR	95%CI	
			Lower	Higher			Lower	Higher
KIF2A high	<0.001	2.072	1.398	3.071	0.818	1.056	0.662	1.686
Age >45 years	0.585	0.897	0.607	1.325	0.479	0.855	0.555	1.318
Male	0.481	1.150	0.780	1.694	0.567	1.135	0.736	1.749
WBC>10*10^9^/L	0.002	2.016	1.301	3.124	0.000	1.045	1.032	1.057
FAB classification								
M1	Ref	-	-	-	Ref	-	-	-
M2	0.860	0.835	0.113	6.197	0.099	0.140	0.014	1.448
M4	0.551	1.840	0.248	13.654	0.330	0.312	0.030	3.248
M5	0.640	1.610	0.219	11.845	0.219	0.234	0.023	2.367
M6	0.450	2.251	0.274	18.466	0.833	0.771	0.069	8.567
Cytogenetics (NK *vs* others)	0.569	1.120	0.758	1.653	0.506	1.200	0.701	2.055
Monosomal karyotype	<0.001	2.981	1.729	5.139	0.650	0.849	0.417	1.725
FLT3-ITD mutation	0.008	1.830	1.168	2.869	0.960	1.015	0.562	1.835
Isolated biallelic CEBPA mutation	0.097	1.643	0.915	2.950	0.364	1.337	0.714	2.503
NPM1 mutation	0.182	0.753	0.496	1.142	0.459	0.797	0.436	1.455
Risk stratification								
Low	Ref	-	-	-	Ref	-	-	-
Intermediate	<0.001	3.584	2.024	6.349	<0.001	5.079	2.493	10.350
High	<0.001	9.550	5.248	17.378	<0.001	23.606	10.430	53.427

HR: hazards ratio; CI: confidence interval; KIF2A: kinesin family member 2A; WBC: white blood cells; FAB classification: French-American-Britain classification systems; NK: normal karyotype; FLT3-ITD: internal tandem duplications in the FMS-like tyrosine kinase 3; CEBPA: CCAAT/enhancer-binding protein α; NPM1: nucleophosmin 1.

**Table 4:** Factors affecting overall survival by Cox proportional hazards regression model analyses.

Parameters	Univariate Cox regression				Multivariate Cox regression			
	P value	HR	95%CI		P value	HR	95%CI	
			Lower	Higher			Lower	Higher
KIF2A high	0.002	2.374	1.368	4.122	0.408	1.323	0.682	2.569
Age >45 years	0.745	0.913	0.528	1.579	0.598	1.182	0.635	2.198
Male	0.351	0.772	0.448	1.330	0.304	0.728	0.398	1.333
WBC>10*10^9^/L	0.044	1.855	1.016	3.387	0.002	1.031	1.012	1.052
FAB classification								
M1	Ref	-	-	-	Ref	-	-	-
M2	0.898	6826.712	0.000	-	0.920	3636.641	0.000	-
M4	0.907	3180.651	0.000	-	0.932	1055.197	0.000	-
M5	0.900	5889.931	0.000	-	0.928	1555.372	0.000	-
M6	0.912	2149.473	0.000	-	0.934	803.242	0.000	-
Cytogenetics (NK *vs* others)	0.494	1.209	0.702	2.082	0.128	1.812	0.843	3.895
Monosomal karyotype	<0.001	4.732	2.324	9.634	0.229	1.790	0.693	4.627
FLT3-ITD mutation	0.157	1.601	0.835	3.071	0.636	0.814	0.347	1.910
Isolated biallelic CEBPA mutation	0.092	1.990	0.894	4.432	0.222	1.743	0.714	4.253
NPM1 mutation	0.046	0.529	0.283	0.990	0.219	0.596	0.261	1.360
Risk stratification								
Low	Ref	-	-	-	Ref	-	-	-
Intermediate	<0.001	14.390	5.883	35.201	<0.001	13.891	4.304	44.840
High	<0.001	4.713	2.015	11.023	0.008	3.829	1.409	10.403

HR: hazards ratio; CI: confidence interval; KIF2A: kinesin family member 2A; WBC: white blood cells; FAB classification: French-American-Britain classification systems; NK: normal karyotype; FLT3-ITD: internal tandem duplications in the FMS-like tyrosine kinase 3; CEBPA: CCAAT/enhancer-binding protein α; NPM1: nucleophosmin 1.

### Comparison of KIF2A expression in human AML cell lines and normal BMMCs

**Figure 4:**
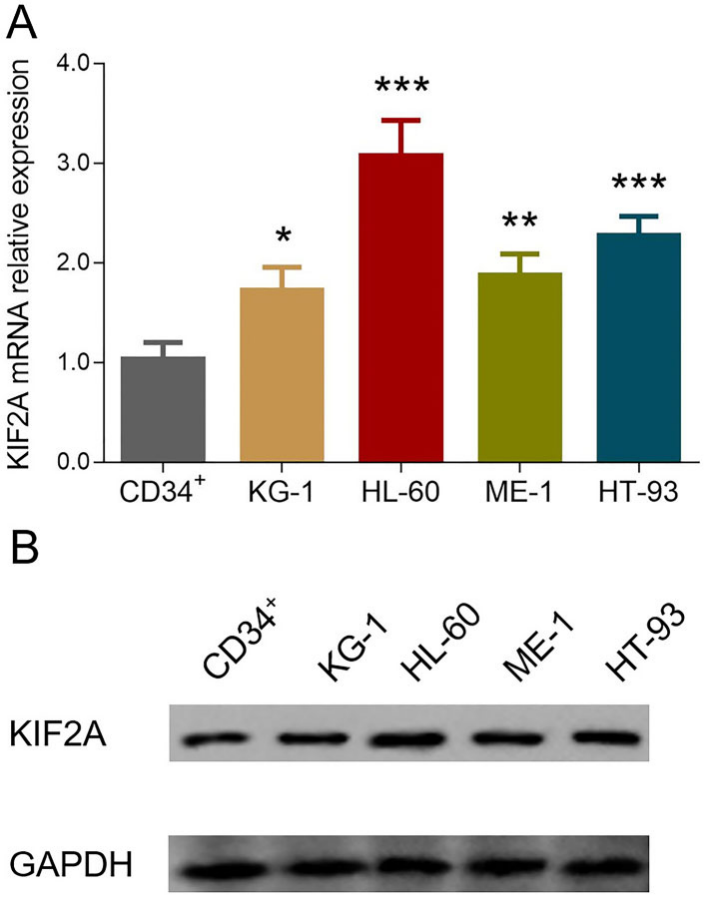
RT-qPCR experiments were carried out in triplicate, and western blot was performed once. **A**, KIF2A mRNA expression in various acute myeloid leukemia (AML) cell lines and CD34^+^ cells; **B**, KIF2A protein expression in various AML cell lines and CD34^+^ cells. Data are reported as means±SD. *P<0.05, **P<0.01, ***P<0.001 compared to CD34^+^ (ANOVA followed by Dunnett *t*-test). KIF2A: kinesin family member 2A.

KIF2A mRNA expression was elevated in KG-1 (P<0.05), HL-60 (P<0.001), ME-1 (P<0.01), and HT-93 cell lines (P<0.001) compared to CD34^+^ cells (Figure 4A). KIF2A protein expression was also increased in KG-1, HL-60, ME-1, and HT-93 cell lines compared to CD34^+^ cells (Figure 4B).

### Effect of KIF2A knockdown on cell proliferation and cell apoptosis in HL-60 cells and KG-1 cells

**Figure 5:**
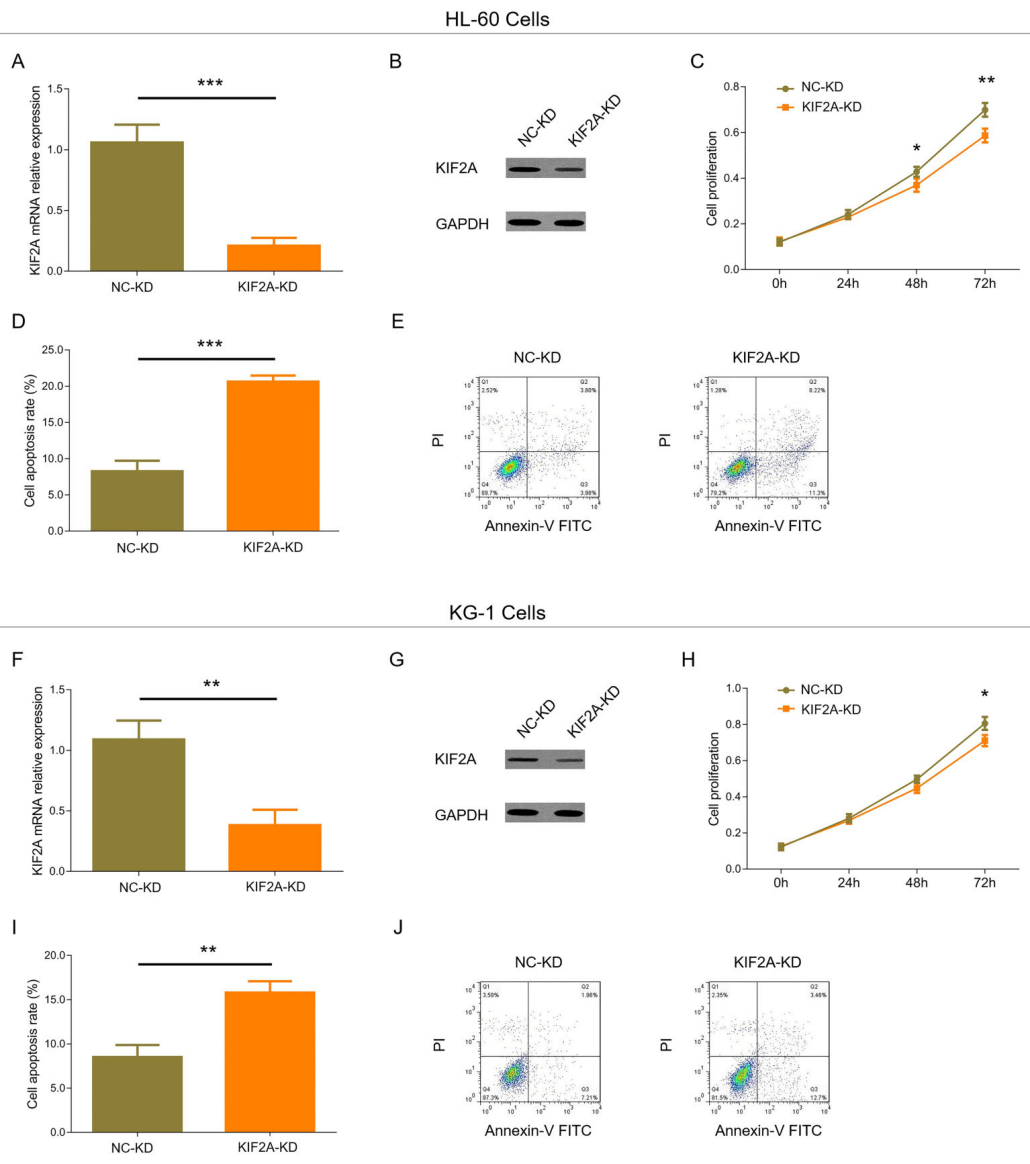
RT-qPCR and CCK-8 experiments were carried out in triplicate, and western blot was performed once. **A**, KIF2A mRNA, **B**, protein expression, **C**, cell proliferation, and **D** and **E**, cell apoptosis in the KIF2A-KD group and the negative control (NC)-KD group in HL-60 cells. **F**, KIF2A mRNA, **G**, protein expression, **H**, cell proliferation, and **I** and **J**, cell apoptosis in the KIF2A-KD group and the NC-KD group in KG-1 cells. Data are reported as means±SD. *P<0.05, **P<0.01, ***P<0.001 (independent samples *t*-test). KIF2A: kinesin family member 2A; KD: knockdown; GAPDH: glyceraldehyde-3-phosphate dehydrogenase; FITC: fluorescein isothiocyanate; PI: propidium iodide.

In our preliminary experiments, we used three shRNAs to knockdown KIF2A, and the one that presented the best efficiency in silencing KIF2A was chosen for further assays. After knockdown KIF2A and knockdown control plasmids were transfected into HL-60 and KG-1 cells, cell proliferation and cell apoptosis were assessed. In HL-60 cells, KIF2A mRNA expression (P<0.001) (Figure 5A) and KIF2A protein expression (Figure 5B) were decreased in the KIF2A-KD group compared to the NC-KD group, indicating successful transfection. Cell proliferation was reduced at 48 (P<0.05) and 72 h (P<0.01) in the KIF2A-KD group compared to the NC-KD group (Figure 5C), whereas cell apoptosis rate was increased at 48 h in the KIF2A-KD group compared to the NC-KD group (P<0.001) (Figure 5D and E). In KG-1 cells, KIF2A mRNA expression (P<0.01) (Figure 5F) and protein expression (Figure 5G) were lower in the KIF2A-KD group compared to the NC-KD group, and cell proliferation was decreased at 72 h in the KIF2A-KD group compared to NC-KD the group (P<0.05) (Figure 5H), but cell apoptosis rate was increased at 48 h in the KIF2A-KD group compared to the NC-KD group (P<0.01) (Figure 5I and J). These data suggested that KIF2A knockdown inhibited cell proliferation but enhanced cell apoptosis in AML.

## Discussion

In this study, we observed that: 1) KIF2A expression was significantly increased in AML patients compared to healthy controls, and correlated with increased WBC level, presence of monosomal karyotype, as well as high risk stratification in AML patients; 2) KIF2A high expression predicted low EFS and OS in AML patients; and 3) KIF2A was overexpressed in various human AML cell lines and its knockdown repressed cell proliferation but enhanced cell apoptosis in AML cells.

KIFs take part in spindle orientation and chromosomal movements during mitosis and cytoskeletal reorganization (8,14-17). As an important member of KIFs, the major function of KIF2A is microtubule depolymerization, which is a critical step for mitotic progression and spindle assembly (9,18-20). Recently, the influence of KIF2A on cancer cells has been investigated (4,7-10). For example, KIF2A knockdown inhibits lymphoma cell proliferation through positively regulating PI3K/AKT signaling pathway in SCCOT Tca8113 cells (7). Furthermore, silencing KIF2A promotes cell apoptosis via inhibiting PI3K/Akt signaling pathway in squamous cell carcinoma of the oral tongue (10). Moreover, silencing KIF2A reduces cell proliferation and migration, enhances cell apoptosis, and induces G2/M phase arrest through inhibiting PI3K/AKT and MAPK/ERK pathways in lung adenocarcinoma cells (11). KIF2A knockdown suppresses cell proliferation, cell invasion, and cell migration through regulating matrix metalloproteinases-2 (MMP-2) activity in human malignant glioma cell lines (4). These data indicate that KIF2A may promote the initiation and progression of some cancers by regulating some enzymes or signaling pathways such as MMP-2, PI3K/AKT, and MAPK/ERK.

Emerging evidence has revealed that KIF2A is overexpressed in tissue samples of several malignancies, such as diffuse large B cell lymphoma (DLBCL), breast cancer, and lung squamous cell carcinoma (7–9). Moreover, the correlation of KIF2A expression with clinical characteristics in cancers is also observed (7). For example, a study reports that KIF2A high expression is associated with elevated Ann Arbor stage and international prognostic index score in DLBCL patients (7). Also, one study reports that KIF2A high expression is correlated with lymph node metastasis and HER2 positive cancer in breast cancer patients (8), and another study shows that KIF2A high expression is associated with increased pathological grade in glioma patients (4). In the present study, we found that KIF2A expression was elevated in AML patients compared to healthy controls, which was in line with the data on AML patient datasets from GEO database, and KIF2A had a good diagnostic value for AML risk. Moreover, KIF2A expression was positively correlated with WBC level, monosomal karyotype possibility, and risk stratification in AML patients. The results found might be due to: 1) KIF2A activation of nuclear factor-kB (NF-kB) pathway via regulating MMP-2, thereby enhancing inflammatory responses and increasing WBC level; and 2) KIF2A regulation of some genes to induce abnormal karyotypes or molecular mutations, such as the monosomal karyotype, and further resulting in high risk stratification.

Some previous studies have revealed the predictive value of KIF2A in various cancers (5,7,8,21). For instance, KIF2A expression has been reported to negatively correlate with OS and be an independent predictive factor for OS in DLBCL, breast cancer, as well as epithelial ovarian cancer patients (8,7,21). These data suggest that KIF2A exhibits good predictive value for poor prognosis in some cancer patients, whereas limited data reveal the predictive value of KIF2A in AML patients. In this study, we observed that KIF2A expression was negatively correlated with EFS and OS in AML patients, possibly because: 1) KIF2A might enhance cell proliferation but inhibit cell apoptosis via regulating various signaling pathways such as MAPK/ERK and PI3K/AKT, therefore it accelerated disease progression and eventually reduced survival time of AML patients (4,7–10); and 2) KIF2A might cause resistance to chemotherapy and further impair treatment efficacy, thereby leading to decreased EFS and OS.

A better understanding on the effect of KIF2A in cancer cells may contribute to explore novel treatment targets. In our study, KIF2A knockdown inhibited cell proliferation and promoted cell apoptosis in both HL-60 and KG-1 cell lines. These data suggested that KIF2A knockdown might serve as an anti-tumor approach by inhibiting cell proliferation and enhancing cell apoptosis in AML cells, which provided indications for further explorations on treatment for AML.

Some limitations of our study were: 1) the sample size was relatively low (N=176), thus there might be a relatively low statistical power; 2) the follow-up duration (median: 17.0 months (range 1.0 to 47.0 months)) was relatively short, therefore the predictive value of KIF2A in long-term treatment outcomes was not assessed; 3) as a single-center study, this study might lack wide representativeness; and 4) therapeutic approaches for AML patients with high KIF2A expression were not suggested in this primary research. Additionally, lack of information about downstream targets of KIF2A was a limitation of our study. Further studies should explore the downstream targets of KIF2A.

In conclusion, KIF2A high expression predicted increased AML risk, and correlated with elevated WBC level, presence of monosomal karyotype, worse risk stratification, shorter EFS, as well as worse OS. Moreover, its knockdown inhibited cell proliferation but enhanced cell apoptosis in AML. These data further confirmed the oncogene role of KIF2A in AML.

## Supplementary Material

Click here to view [pdf].
